# Reconstructive spectrometers taper down in price

**DOI:** 10.1038/s41377-023-01190-7

**Published:** 2023-06-07

**Authors:** Xiaoqi Cui, Yi Zhang, Andreas C. Liapis, Zhipei Sun

**Affiliations:** QTF Centre of Excellence, Department of Electronics and Nanoengineering, Aalto University, Tietotie 3, FI-02150 Espoo, Finland

**Keywords:** Optical spectroscopy, Fibre optics and optical communications

## Abstract

The development of a low-cost compact reconstructive spectrometer paves the way towards portable pm-resolution spectroscopy.

Optical spectroscopy has long been an indispensable tool for both industry and scientific research. For many emerging applications, such as in the rapidly growing market of wearable electronics, portability is of paramount importance. For example, there is a need for cheaper and smaller spectrometers to be integrated into compact devices, such as smartwatches that monitor our biosignals, or portable analyzers that can detect counterfeit pharmaceuticals. Perhaps in the future, we might even be able to detect invisible food spoilage using our phones or smart glasses. Although significant efforts have been made to the development of many different classes of spectrometers^1,2^, in the race towards low-cost, stable, compact, fast, power-efficient, and high-resolution optical spectroscopy, reconstructive spectrometers are poised to take the lead.

Traditional spectrometers rely on dispersive elements (typically a grating or a prism) to separate incident light into its constituent colours; the spectral content is then read by a one-dimensional detector array. Generally speaking, spectral resolution scales with linear dimensions, so these spectrometers tend to be bulky. By contrast, reconstructive spectrometers are designed such that each input wavelength produces a complex but unique pattern as the output signal. After a training step in which the response of the device to known spectra is characterised, it is feasible to extract the frequency content of unknown spectra from the device’s response to illumination using a computational algorithm.

Reconstructive spectrometers can be significantly more compact than benchtop designs without sacrificing performance. The smallest footprints—on the order of a few tens of micrometres—are seen on devices whose output signal is electrical^3–6^. Such devices behave as tunable energy filters, typically as a function of the applied electrical signal. However, thus far, these ultra-miniaturised spectrometers face various issues regarding their reproducibility, stability, and operation speed^6^. The highest spectral resolutions, on the other hand, are obtained by devices that rely on multimode interference to create wavelength-dependent spatial patterns^7–9^. For example, spectral information has previously been extracted from how light leaks from a multimode fiber taper^10^. While interference regions of just a few hundred microns in length are sufficient to obtain picometre spectral resolution, external optics are required to image and acquire the spatial patterns, hampering the portability of these spectrometers.

In the current issue of eLight, Yaoguang Ma and colleagues from Zhejiang University, China, report on a compact self-contained reconstructive spectrometer based on multimode interference by placing a tapered optical fiber over a CMOS (Complementary Metal Oxide Semiconductor) image sensor^11^. The entire functional unit has a size of ~1 mm^2^. The working principle of their spectrometer is shown in Fig. 1. The input light is first coupled from a single-mode fiber to a multimode fiber, exciting multiple transverse optical modes. The fiber is then tapered down such that the multiple modes leak out in a complex spatial pattern. Because different wavelengths have different mode indices, each wavelength’s spatial patterns are unique. The leaked light is directly captured by the CMOS image sensor and analysed by a lightweight vision transformer network. This neural network can reconstruct the input spectrum with a resolution down to ~1 pm. One single sensor shot (a few tens of milliseconds and microwatts) is enough to gather sufficient information to derive the spectrum. Remarkably, they also showed that many fiber tapers could be placed on the same sensor enabling the simultaneous acquisition of multiple spectra for spectral imaging.

**Fig. 1:**
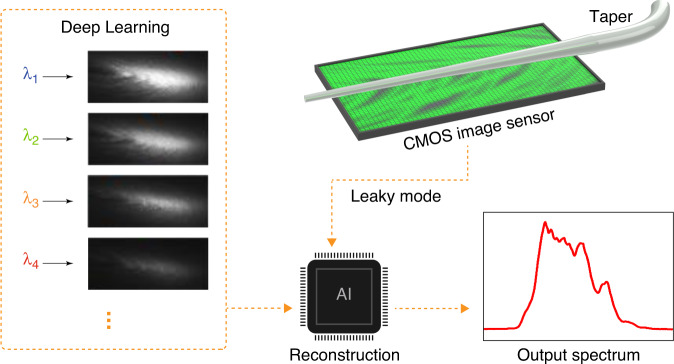
The working principle of the self-contained reconstructive spectrometer with multimode interference. The patterns in the deep learning box are adapted with permission from ref. ^11^

A major advantage of this approach is that the optical patterns carrying spectral information are read out by a commercial CMOS image sensor, which is a mature technology. This spectrometer can therefore be integrated with portable devices; no additional read-out electronics need to be developed. In addition to being portable, this spectrometer is cheap to construct. The team provides a cost-effective (less than ~$15) and fabrication-friendly design (the fiber taper can even be drawn with bare hands and an alcohol lamp).

This technology is expected to shine in some applications where portability is required.
